# Propofol-induced frontal aEEG changes in children during deep procedural sedation

**DOI:** 10.3389/fneur.2025.1566864

**Published:** 2025-06-30

**Authors:** Luisa Paul, Eva Tschiedel, Anna Daniels, Pia Brensing, Carolina A. Joist, Constantin M. Joist, Sandra Greve, Ursula Felderhoff-Müser, Christian Dohna-Schwake, Nora Bruns

**Affiliations:** ^1^Department of Pediatric Cardiology/Congenital Cardiology, Heidelberg University Medical Center, Heidelberg, Germany; ^2^Department of Pediatrics I, Neonatology, Pediatric Intensive Care Medicine, Pediatric Neurology, and Pediatric Infectious Diseases, University Hospital Essen, University of Duisburg-Essen, Essen, Germany; ^3^C-TNBS, Centre for Translational Neuro-and Behavioural Sciences, University Hospital Essen, University of Duisburg-Essen, Essen, Germany

**Keywords:** amplitude-integrated EEG (aEEG), children, procedural sedation, propofol, midazolam

## Abstract

**Background:**

Amplitude-integrated EEG (aEEG) is an important neuromonitoring tool in paediatric critical care, but effects of agents used for procedural sedation on aEEG patterns are not understood. The aim of this study was to explore the correlation between deep procedural sedation and modifications in aEEG amplitudes in children without cerebral pathologies.

**Methods:**

In this prospective observational study, 165 children aged 6 months to 17.9 years undergoing procedural sedation with propofol and premedication with midazolam were monitored using frontal aEEG (Fp1, Fp2, FpZ according to the 10–20 system). Sedation depth was assessed using the Comfort Score (CS).

**Results:**

The median patient age was 8.8 years (interquartile range 3.9–14.0), with a median procedure duration of 19 min. A total of 1,464 paired observations of CS and amplitude measurements were analyzed. The lower amplitude showed a moderate negative correlation with CS (deeper sedation associated with higher amplitude), increasing by 1.4 μV per one-point decrease in CS with variations between age groups. The upper amplitude remained largely unchanged during deep sedation, whereas the bandwidth narrowed. The lower amplitude increased from baseline by a median of 6.5 μV (37.9% relative increase), with variations across age groups.

**Conclusion:**

Deep procedural sedation with propofol primarily affects the lower amplitude of frontal aEEG, with age-dependent variations. These findings advance the understanding of propofol-induced aEEG changes in neurologically healthy children, which may enhance bedside aEEG interpretation in paediatric patients.

## 1 Introduction

In paediatric critical care, the assessment of neurological function in critically ill or sedated children is challenging (1). Since clinical neurological evaluation is impossible the use of different neuromonitoring techniques are required. Among these, full channel continuous electroencephalography (EEG) is the most common (2).

One of the employed techniques to facilitate the interpretation of EEG in the pediatric intensive care unit (PICU) is the mathematical transformation of the raw EEG into quantitative EEG (qEEG) panels (3, 4). One common transformation is amplitude-integrated EEG (aEEG), which focuses on the amplitude’s height during a given time period (5). aEEG is a bedside technology that provides continuous monitoring of brain function (6). It has proven a useful tool for the early seizure detection, that might be missed in critically ill children and the background pattern of aEEG can be associated with neurological outcome in neonatal and pediatric neurocritical illness (2, 7–10). Paediatric critical care teams across Europe are familiar with the application and interpretation of this technique as it is an indispensable tool for diagnostics and therapy monitoring in asphyxiated newborns in the NICU and can serve as complementary or bridging technology to full channel (continuous) EEG in the PICU.

A circumstance complicating (a) EEG interpretation in the PICU is the frequent requirement of neuroactive substances in critically ill children and their interaction with acute (neuro-)critical illness (11). For example, propofol administration can result in amplitude rise or suppression, depending on the concomitant illness and accompanying drugs (12). However, the specific effects of propofol on aEEG patterns in neurologically healthy children remain poorly understood, limiting the ability to distinguish between sedation-induced changes and pathological alterations in clinical practice. Examining the electrocortical activity of neurologically healthy children during elective or semi-elective procedural sedation presents an opportunity to investigate sedation-induced aEEG changes without confounding cerebral illness.

This study is a *post-hoc* analysis of a prospective observational study on the correlation of the Narcotrend index with clinical sedation depth during deep procedural sedation with propofol in children (13). The *post-hoc* analysis was conducted to investigate the association of frontal aEEG with clinical sedation depth.

## 2 Methods

This is a *post-hoc* analysis of a previously published prospective study on the correlation of the Narcotrend index with the comfort score (CS) during procedural sedation (13).

We prospectively included children between 6 months and 17.9 years of age undergoing procedural propofol sedation in a tertiary PICU of the University Hospital Essen between October 2020 and December 2022. Exclusion criteria were underlying neurologic diseases potentially impairing Comfort scale scoring, known EEG abnormalities, prior participation in this study, and anticipated use of ketamine or remifentanil. Patients who unexpectedly received ketamine or remifentanil during the sedation were retrospectively excluded.

Eligible procedures were endoscopies, bronchoscopies, biopsies, and punctures including drain placements. Shortly after the initiation of the study, routine sedation regimes for muscle biopsies and bronchoscopies were changed to remifentanil + propofol, resulting in secondary exclusion of these procedures.

### 2.1 Sedation

Sedation was performed by experienced paediatric intensivists (AD, CD-S, ET) in the PICU and followed international guidelines (14, 15). According to our standard procedural sedation regime, intravenous (i.v.) midazolam (0.05 mg/kg, maximum 2 mg) was administered as premedication before placement of EEG electrodes (section EEG recording), followed by an i.v. induction bolus of propofol (1 mg/kg) and continuous infusion of propofol (10 mg/kg/h). The sedation level was optimized by clinical assessment (CS target range 11–14) via administration of propofol boli (1 mg/kg) or adjustment of the continuous infusion rate as required to reach the CS target range. Sedation was immediately stopped at the end of the procedure. All patients received oxygen via a nasal cannula throughout the sedation.

### 2.2 Clinical measurement of sedation depth

Sedation depth was assessed using the CS, our standard for the assessment of sedation depth during procedural sedation. The Comfort Score is a clinical assessment tool used to evaluate sedation, pain, and distress in critically ill children, incorporating six behavioral domains: alertness, calmness-agitation, respiratory response, crying, physical movement, and muscle tone/facial tension. Each domain is scored from 1 to 5, with total scores ranging from 6 (indicating oversedation) to 30 (indicating full wakefulness or inadequate sedation) (16).

From the beginning of the sedation, besides responses to intervention-related stimuli, CS was recorded every 5 min. After cessation of the procedure, a standardized painful stimulus was applied to the sternum every 5 min until eye-opening. As during the procedure, the reaction to the stimulus was recorded as part of the CS assessment.

### 2.3 Clinical documentation

The CS, propofol infusion rate was documented manually at the beginning of sedation and every 5 min until eye-opening by medical doctorate students that were not involved in the sedation or the procedure. Propofol bolus application and adverse events were documented whenever they occurred. All protocols were then entered into Excel sheets, including pseudonyms and time stamps.

### 2.4 EEG recording

**Figure 1:**
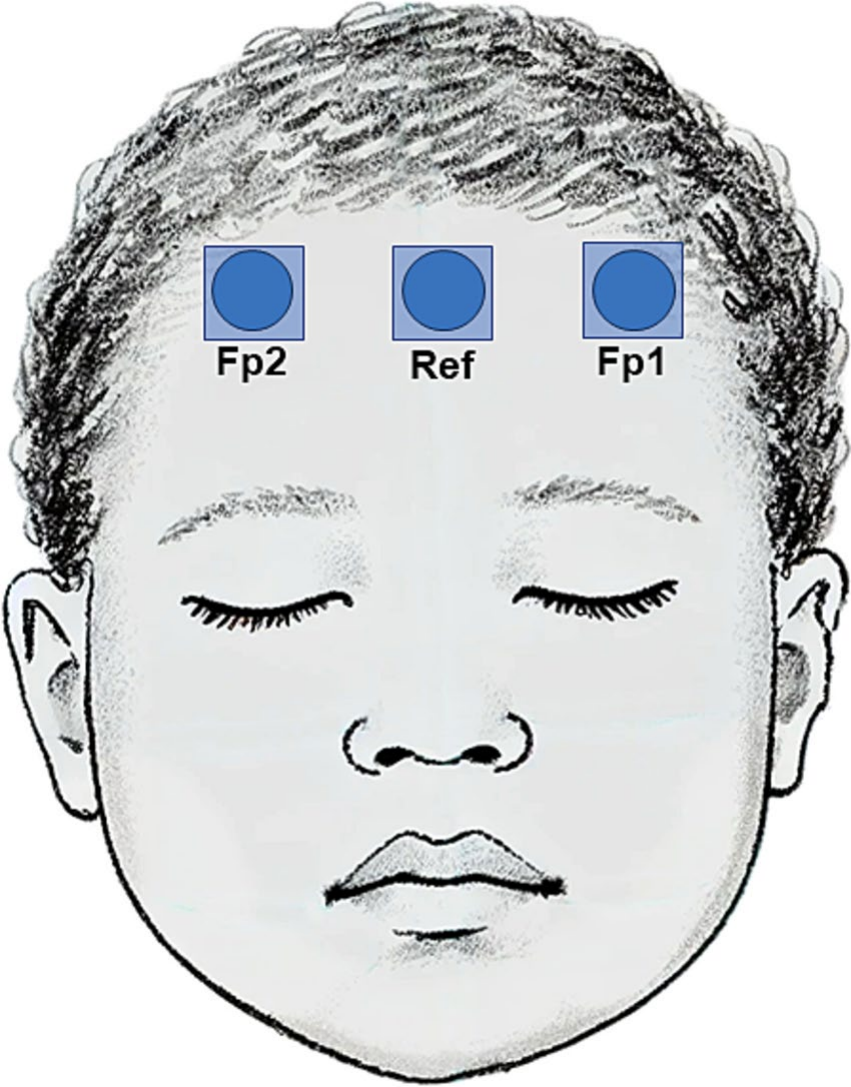
Schematic illustration of aEEG electrode positions on the forehead of a child. Three hydrogel electrodes were placed in the Fp1, Fp2, and FpZ position according to the international 10–20 system.

EEGs were recorded using BrainTrend 2.0 monitors (MT Monitortechnik, Bad Bramsted, Germany) using the manufacturer’s set up for intraoperative monitoring. Three hydrogel electrodes used in clinical routine care were placed on the forehead in the Fp1, Fp2, and FpZ position according to the international 10–20 system (Figure 1). Skin preparation was performed with OneStep EEG Gel Abrasiv plus® (H + H Medizinprodukte, Münster, Germany) until impedance values were <10 kΩ.

Because the recorded EEGs were not part of the clinical routine, all recordings were pseudonymized without patient-identifying information contained in the EEG file. Vital sign monitoring was carried out according to the clinical routine.

### 2.5 aEEG processing

EEGs were processed by the manufacturer of the BrainTrend monitor for the purpose of the post-hoc analysis. Upper and lower amplitudes were calculated for each 5 s epoch by averaging the highest and lowest values per epoch. In addition, the read-outs contained the pseudonym and information on automated artifact detection and electromyogram (EMG) detection for each time stamp.

### 2.6 Data cleaning of aEEG tracings and merging with clinical data

For statistical analyses, the EEG read-outs were cleaned by removing observations with artifacts identified by the manufacturer’s built-in artifact detection, which includes EMG detection, and suspected electrode dislocation (upper amplitude measurements < 5 μV, lower amplitude < 3 μV, bandwidth < 5 μV, Narcotrend index non-classifiable). No manual review of the raw-EEG was conducted.

The cleaned EEG read-outs were matched with the manual records for each patient and each time stamp, creating paired observations that were used for further analyses. Because there were more observations per EEG read-out than by manual recording, the number of paired observations was determined by the number of manual clinical observations per patient. To avoid distortion of results from muscle artifacts in partially or fully awake patients, correlation and regression analyses included only paired observations with a CS ≤ 20.

### 2.7 Statistical analyses

Continuous variables are presented as mean if evenly distributed and as median if skewed. Discrete variables are summarized as counts and relative frequencies. Stratified analyses were conducted by age groups (< 3 years, 3–5 years, 6–11 years, 12–17 years).

The Pearson correlation coefficients (PCC) between CS and the upper and lower aEEG amplitudes and bandwidth were calculated for the entire cohort, stratified by age, and within each individual. The median and interquartile range for intraindividual PCCs were calculated. The strength of correlation was interpreted as suggested in the context of biomedical research (17).

To assess the shape of the association between amplitudes and CS we used penalized b-spline regression. Because the splines were almost completely linear, we then applied linear regression to calculate the change of aEEG amplitudes per unit change of the CS. Because repeated measurements within one individual were not independent, we used hierarchical modelling with the individual patient as a random effect and age as a fixed effect.

Next, individual trajectories of aEEG amplitudes during sedations were plotted and visually analyzed. Because these trajectories tended to show interindividual differences of baseline amplitude values with varying increase of the upper and lower amplitudes during sedation, additional analyses were performed to assess the absolute and relative change from the individual baseline during sedation. For this purpose, amplitude values for each minute were averaged. Next, the first 5 min of each recording were extracted and amplitude values averaged. These values were compared to the amplitude values observed at the lowest CS during each recording. For the lowest CS value, the absolute in μV and relative % change from baseline in percent was calculated for each patient.

All statistical analyses were performed using SAS Enterprise Guide Version 8.3 (SAS Institute Inc., Cary, NC, USA). Figure 1 was created using DALL-E3 (Open AI, San Francisco, CA, USA) from the prompt of a child face. The AI drafted image was then edited in Corel PHOTO-PAINT 2021 (Corel Corporation, Ottawa, Canada) and Microsoft Office PowerPoint 16.9 (Microsoft Corporation, Redmont, WA, USA). All other figures were produced using SAS Enterprise Guide and Microsoft Office PowerPoint.

### 2.8 Ethics approval

The study was approved by the local ethics committee (19-8728-BO, 21-10306-BO). Written informed consent was obtained from the legal guardians of all included patients.

## 3 Results

**Figure 2:**
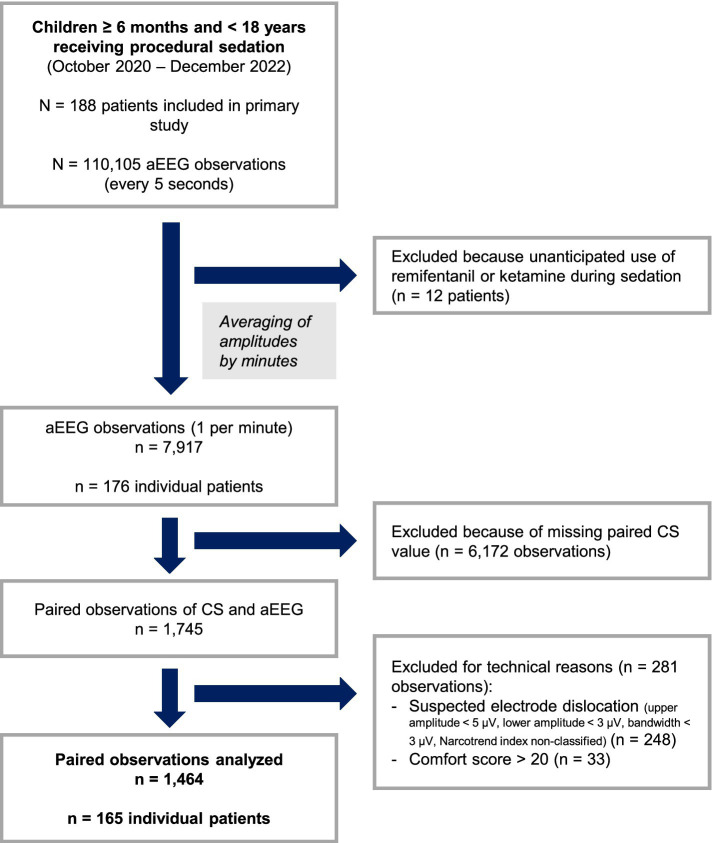
Flowchart of data processing.

Of 176 patients included in the original study, 165 had eligible EEG recordings after completion of the data cleaning procedure (Figure 2). A total of 1,464 paired observations consisting of CS and amplitude measurements were analyzed, corresponding to 8.9 ± 3.5 observations per patient. The median patient age was 8.8 years (IQR 3.9–14.0) with a median weight of 28.0 kg (IQR 17.0–50.0) (Table 1). The most frequently performed procedures were biopsy (liver, kidney, skin, muscle, thyroid), puncture (lumbal, pleural drainage, ascites drainage, bone marrow, joint), and esophagogastroduodenoscopy, placement of percutaneous gastroenterostomy, transoesophageal echocardiography with a median duration of 19 min.

**Table 1:** Clinical and periprocedural information.

	Overall	0–2 years	3–5 years	6–11 years	12–17 years
*N* (%)	165 (100%)	16 (9.7%)	45 (27.3%)	43 (26.0%)	61 (37.0%)
Procedure					
Esophagogastroduodenoscopy, placement of percutaneous gastroenterostomy, transesophageal echocardiography; *n* (%)	45 (27.3%)	4 (2.4%)	14 (8.48%)	13 (7.88%)	14 (8.48%)
Colonoscopy, rectoscopy; *n* (%)	5 (3.0%)	0 (0.0%)	2 (1.21%)	2 (1.21%)	1 (0.61%)
Placement of pH-metry probe; *n* (%)	3 (1.8%)	1 (0.61%)	0 (0.00%)	2 (1.21%)	0 (0.00%)
Bronchoscopy; *n* (%)	6 (3.6%)	4 (2.42%)	0 (0.00%)	2 (1.21%)	0 (0.00%)
Biopsy (liver, kidney, skin, muscle, thyroid); *n* (%)	72 (43.6%)	4 (2.42%)	16 (9.70%)	15 (9.09%)	37 (22.42%)
Puncture (lumbal, pleural drainage, ascites drainage, bone marrow, joint); *n* (%)	48 (29.1%)	5 (3.03%)	13 (7.88%)	14 (8.48%)	16 (9.70%)
Catheter placement or removal (central venous catheter, Shaldon, Broviac); *n* (%)	14 (8.5%)	1 (0.61%)	4 (2.42%)	4 (2.42%)	5 (3.03%)
Multiple procedures; *n* (%)	21 (12.7%)	3 (18.8%)	2 (4.4%)	7 (16.3%)	9 (14.8%)
Duration of sedative administration [min], median (IQR)	19.0 (14.0–25.0)	24 (20.0–30.0)	16 (14.0–20.0)	17 (12.0–28.0)	20 (14.0–30.0)
Time until eye-opening [min], median (IQR)	16.0 (10.0–3.0)	26.5 (21–33.5)	20.0 (14.0–25.0)	17.0 (10.0–23.0)	13.0 (7.0–16.0)
Propofol dose					
Total [mg/kg], median (IQR)	17.0 (12.9–23.2)	19.0 (14.9–23)	22.4 (15.7–27.3)	19.1 (14.0–23.3)	12.9 (10.2–17.1)
Induction dose via bolus application [mg/kg], median (IQR)	2.5 (1.8–4.1)	3.9 (2.4–4.3)	2.4 (1.9–3.6)	2.6 (1.7–4.1)	2.4 (1.7–3.6)
Maintenance dose via syringe pump ± intermittent bolus application [mg/kg], median (IQR)	5.6 (4.1–7.7)	7.4 (6.0–8.9)	6.3 (5.2–7.9)	6.0 (4.4–7.2)	4.3 (3.1–5.9)

IQR, interquartile range.

**Figure 3:**
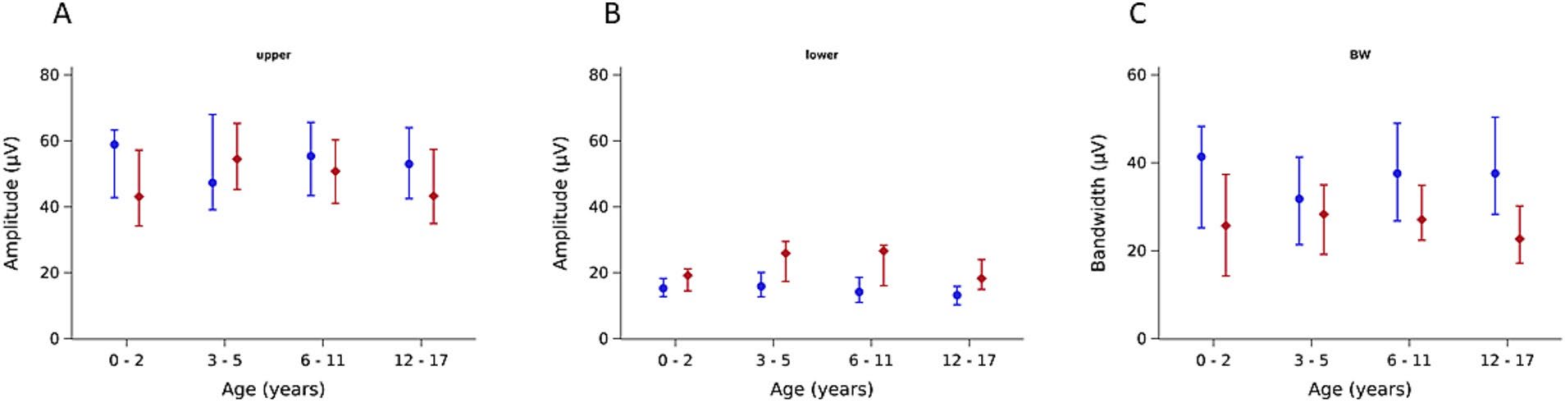
Baseline amplitudes (blue) and during deep sedation (red) by age groups (median and interquartile range). **(A)** Upper Amplitude, **(B)** Lower Amplitude, **(C)** Bandwidth.

We observed an age-dependency of amplitude in heights. The upper amplitude showed an increase in the amplitude height up to the age of 5 years, followed by a slight decrease until the age of 17.9 years [52.5 μV (IQR 40.4–64.4)]. The upper amplitude during the deepest sedation was lower compared to the baseline, but also age-dependent. The baseline of the lower amplitude showed no relevant change with increasing age [14.4 μV (IQR 11.3–18.6)]. The amplitude height was higher during the deepest sedation compared to the baseline. We also observed an age-dependency of the lower amplitude during deepest sedation with an increase of the amplitude height up to 5 years of age and after that a slight decrease [21.2 μV (IQR 15.8–28.0)]. The bandwidths (baseline and deep sedation) were higher in small infants (0–2 years), decreased between 3 and 5 years and then increased slightly. Amplitude height was lower during deep sedation compared to the baseline (Figure 3; Table 2).

**Table 2:** Correlation and regression coefficients.

	Overall *n* = 165	0–2 years *n* = 16	3–5 years *n* = 45	6–11 years *n* = 43	12–17 years *n* = 61
Number of paired observations used for analysis	1,464	148	401	365	551
Upper amplitude					
Baseline amplitude, median (IQR)	52.5 (40.4–64.4)	58.85 (42.84–63.26)	47.33 (39.12–67.96)	55.36 (43.36–65.61)	52.99 (42.5–63.96)
Amplitude during deepest sedation, median (IQR)	49.9 (36.7–59.7)	43.09 (34.21–57.18)	54.48 (45.25–65.30)	50.83 (41.14–60.31)	43.34 (34.93–57.44)
Absolute change from baseline (μV), median (IQR)	−1.2 (−16.8–8.7)	−8.13 (−22.47–5.98)	2.37 (−9.70–15.63)	0.75 (−13.22–14.77)	−5.95 (−19.51–2.77)
Relative change from baseline (%), median (IQR)	−2.4 (−29.2–18.1)	−18.49 (−37.07–17.99)	5.43 (−17.65–40.23)	1.68 (−24.44–25.80)	−10.11 (−34.48–6.24)
Overall correlation of CS and amplitude, PCC (95% CI)	−0.02 (0.02 – −0.07)	0.02 (−0.15 to −0.18)	−0.08 (−0.17 to −0.02)	−0.05 (−0.16–0.05)	0.00 (−0.08–0.09)
Intraindividual correlation of CS and amplitude, median (IQR)	−0.07 (−0.43–0.23)	−0.06 (−0.32–0.21)	−0.10 (−0.42–0.27)	−0.11 (−0.56–0.21)	−0.07 (−0.43–0.16)
Regression slope (change of amplitude (μV) per one point increase of comfort score), estimate (95% CI)	−0.4 (−0.9–0.0)	−0.7 (−2.6–1.1)	−0.6 (−1.3–0.2)	−1.0 (−2.0–0.1)	0.1 (−0.6–0.7)
Lower amplitude					
Baseline amplitude (μV), median (IQR)	14.4 (11.3–18.6)	15.31 (12.79–18.33)	15.93 (12.71–20.05)	14.19 (11.03–18.55)	13.16 (10.27–15.88)
Amplitude during deepest sedation (μV), median (IQR)	21.2 (15.8–28.0)	19.20 (14.46–21.22)	25.89 (17.37–29.53)	26.62 (16.07–28.39)	18.28 (14.99–23.99)
Absolute change from baseline (μV), median (IQR)	6.5 (0.5–12.2)	2.58 (−2.58–6.86)	6.73 (1.92–13.75)	7.52 (−0.14–16.70)	6.61 (0.73–11.03)
Relative change from baseline (%), median (IQR)	37.9 (3.6–99.6)	17.22 (−9.86–43.76)	42.81 (10.01–96.13)	38.87 (−0.88–132.23)	54.33 (6.0–100.73)
Overall correlation of CS and amplitude, PCC (95% CI)	−0.30 (−0.26 to −0.34)	−0.20 (−0.35 to −0.04)	0.28 (−0.37 to −0.19)	−0.35 (−0.433 to −0.25)	−0.35 (−0.43 to −0.28)
Intraindividual correlation of CS and amplitude, median (IQR)	−0.46 (−0.70 to −0.21)	−0.20 (−0.47–0.15)	−0.44 (−0.72 to −0.07)	−0.48 (−0.70 to −0.21)	−0.52 (−0.74 to −0.36)
Regression slope (change of amplitude (μV) per one point increase of comfort score), estimate (95% CI)	−1.4 (−1.5 to −1.2)	−0.9 (−1.4 to −0.3)	−1.3 (−1.6 to −0.9)	−1.7 (−2.1 to −1.3)	−1.3 (−1.6 to −1.1)
Bandwidth					
Baseline amplitude, median (IQR)	36.9 (26.8–90.5)	41.43 (25.21–48.31)	31.75 (21.40–41.27)	37.62 (26.78–48.98)	37.62 (28.32–50.37)
Amplitude during deepest sedation, median (IQR)	25.7 (19.3–90.2)	25.67 (14.28–37.41)	28.30 (19.22–34.95)	27.05 (22.38–34.94)	22.70 (17.20–30.20)
Absolute change from baseline (μV), median (IQR)	−9.2 (−20.1–53.7)	−10.24 (−17.10 to −4.54)	−4.09 (−12.53–3.83)	−9.02 (−19.65–1.67)	−11.55 (−25.36 to −4.06)
Relative change from baseline (%), median (IQR)	−25.4 (−45.3–2.1)	−27.84 (−44.16 to −14.71)	−14.19 (−37.13–13.80)	−23.70 (−40.05–4.57)	−33.12 (−55.27 to −14.95)
Overall correlation of CS and amplitude, PCC (95% CI)	0.13 (0.08–0.18)	−0.20 (−0.35 to −0.04)	−0.28 (−0.37 to −0.19)	−0.35 (−0.43 to −0.25)	−0.35 (−0.43 to −0.28)
Intraindividual correlation of CS and amplitude, median (IQR)	0.10 (−0.19–0.54)	0.00 (−0.20–0.21)	0.09 (−0.22–0.49)	0.10 (−0.17–0.54)	0.18 (−0.21–0.56)
Regression slope (change of amplitude (μV) per one point increase of comfort score), estimate (95% CI)	0.9 (0.6–1.3)	0.2 (−1.4–1.6)	0.7 (0.1–1.3)	0.8 (−0.0–1.6)	1.4 (0.9–1.9)

CI, confidence interval; IQR, interquartile range.

The overall PCC for the lower amplitudes showed a moderate negative correlation, whereas the PCC for the upper amplitude was close to zero and for the bandwidth slightly positive (Table 2). Age group analysis showed that the negative correlation (PCC) between CS and the lower amplitude became positive with increasing age, rising from −0.18 in the youngest to +0.34 and +0.33 in the highest age groups (Table 2). Intraindividual PCCs were higher than when analyzing the entire cohort (Table 2).

**Figure 4:**
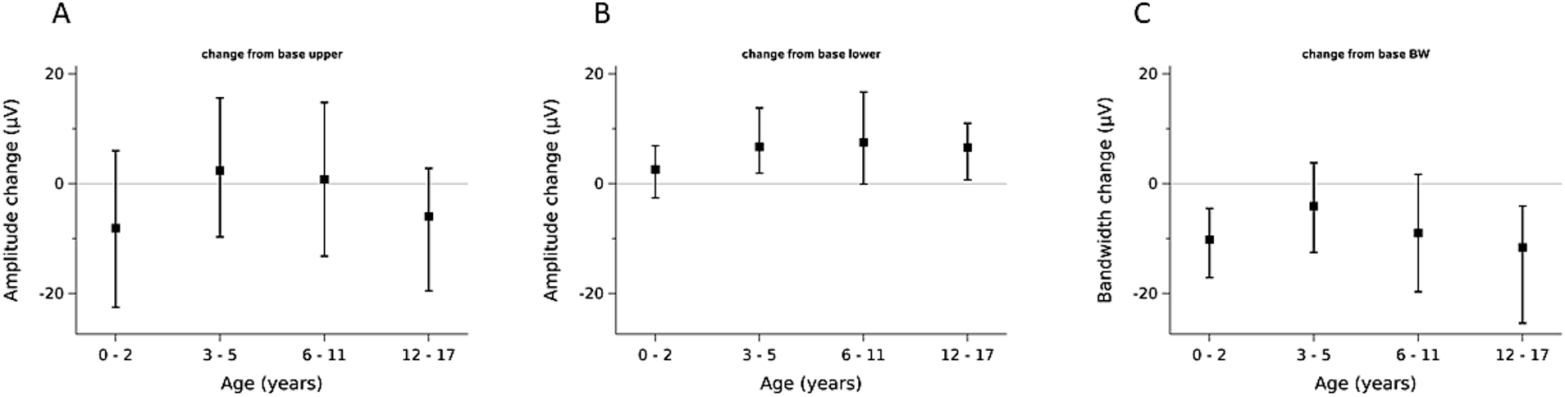
Amplitude changes from baseline to deep sedation by age groups (median and interquartile range). **(A)** Upper Amplitude, **(B)** Lower Amplitude, **(C)** Bandwidth.

Regression analysis showed that the lower amplitude increased −1.4 μV per one point decrease of the CS (= deeper sedation), with differing slopes according to age groups (Figure 3). For the upper amplitude in the overall cohort and age groups almost no change was observed at different CS values, whereas the bandwidth became slightly broader with lowering CS values (Figure 4).

**Figure 5:**
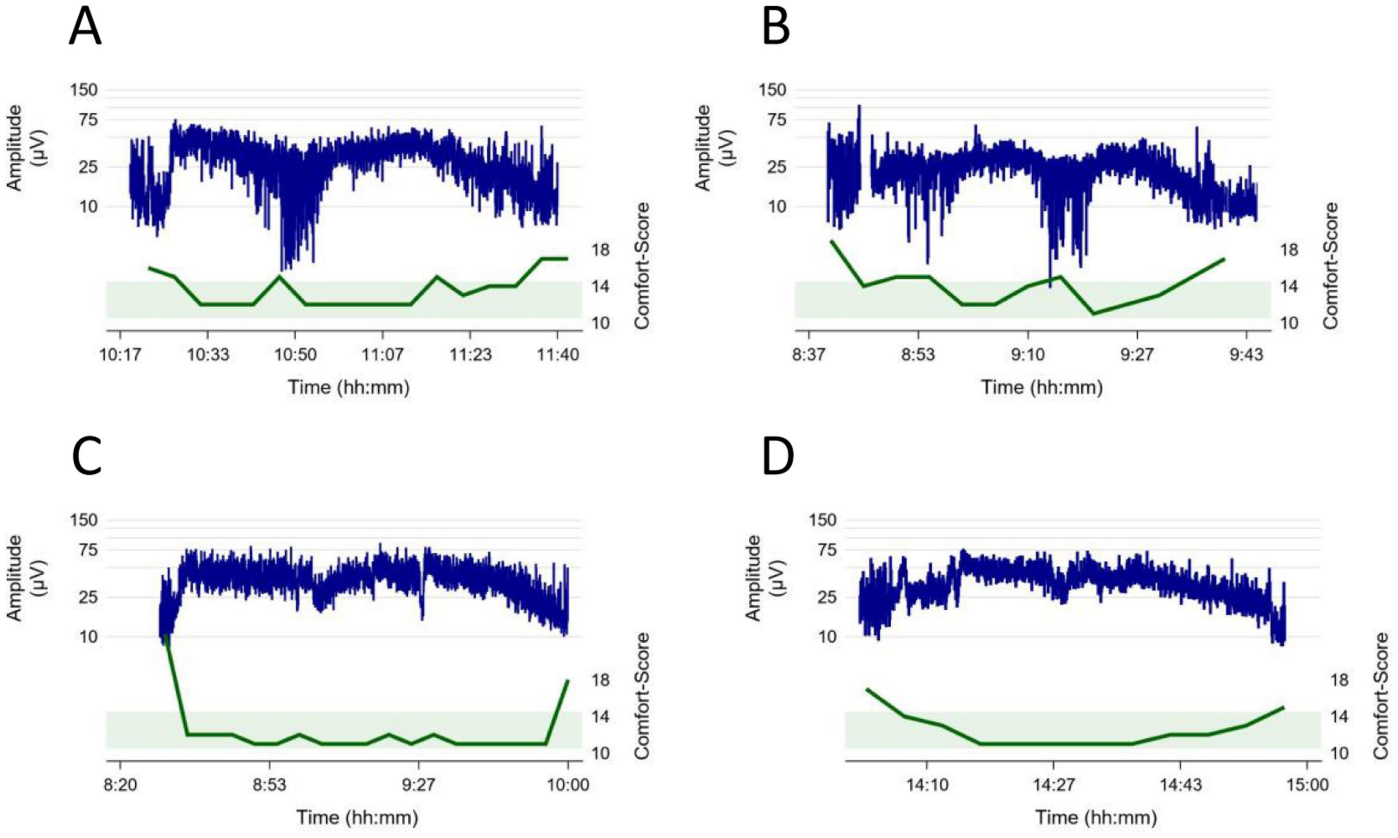
Examples of intraindividual Comfort Score (green) and aEEG (blue) trajectories throughout the sedation. The green boundary illustrates the ideal Comfort score between 10 and 14 during sedation. **(A)** 14-year-old patient, procedure: lumbar puncture, bone marrow aspiration, intrathecal chemotherapy, **(B)** 12-year-old patient, procedure: esophagogastroduodenoscopy, liver biopsy, **(C)** 2-year-old patient, procedure: placement of a central venous catheter and abdominal ultrasound **(D)** 1-year-old patient, procedure: esophagogastroduodenoscopy, bronchoscopy, placement of pH-metry probe.

Investigating individual trajectories, we observed that the individual baseline of the lower amplitude was lower at the beginning of the sedation than during deep sedation (Table 2; Figure 3). For the upper amplitude change were less consistent, with the youngest and the oldest age groups experiencing amplitude decreases and the middle age groups slight amplitude increases (Table 2; Figure 3). Examples of intraindividual CS and aEEG trajectories throughout the sedation are displayed in Figure 5.

## 4 Discussion

This study found aEEG amplitude changes of neurologically healthy children undergoing elective or semi-elective deep procedural sedation with propofol. The lower amplitudes increased during sedation, whereas the upper amplitudes stayed largely unaffected and bandwidths narrowed. Subgroup analyses revealed that the strength of the induced amplitude changes varied with age. These results highlight the interplay between sedation depth and age in amplitude-integrated EEG.

Previous studies investigating the association of aEEG amplitudes with sedation neonate and paediatric intensive care patients found diverging results, driven by varying patient groups and investigated substances. It has been observed that minimum and maximum amplitudes of parietal aEEG became higher after induction in children undergoing anaesthesia with inhaled gases and opioids (18). In newborns undergoing cardiac surgery, postoperative use of midazolam was associated with transient loss of sleep wake-cycling, whereas the use of fentanyl induced long-lasting severe amplitude suppression in some infants (19). Giordano et al. found increased discontinuity of the aEEG background according to the sedation level but only rarely burst suppression in neonates receiving midazolam ± opiates (20). High doses of sedatives and anticonvulsants combined can induce burst suppression pattern in infants and children, whereas lower sedative doses or ketamine administration increase aEEG amplitudes in children (12). In adult patients after cardiac arrest, higher aEEG amplitudes and lower suppression ratios were associated with survival and that EEG amplitudes increased during the daily wake-up trial (21).

During anesthesia, background patterns in neonates and preterm infants become more discontinuous and can even reach flat trace, depending on postmenstrual age and sevoflurane concentration (22). In older infants and children, EEG monitoring during anesthesia showed that amplitudes were higher during deeper anesthesia stages as measured by the Narcotrend index and also observed age dependent effects (23). To avoid too deep anesthesia with burst suppression or flat trace, EEG monitoring can aid in finding the sufficient propofol dose during transition from sevoflurane to intravenous anesthesia (24). The present study mainly aligns with the here-cited studies on aEEG during anesthesia in infants and children, as the study populations were neurologically healthy and received elective induction of coma. Even though the hypnotic depth in our study was lower, we observed the same direction of amplitude changes induced by sedation as the studies by Schultz and Dennhardt. This study broadens the existing body of evidence on aEEG changes during anesthesia by adding insights on deep to intermediate sedation, with findings that align with previous research and also play a role in sedated PICU patients.

While our findings are derived from neurologically healthy children undergoing elective procedures, they have important implications for pediatric intensive care practice where the interplay between sedation, underlying neurological pathology, and organ dysfunction makes aEEG interpretation highly complex. Previous research has demonstrated that normal aEEG amplitudes are associated with good outcomes in critically ill children, while bilateral amplitude suppression predicts mortality and functional decline with approximately 70% specificity (25). Abnormal EEG background patterns, including discontinuous or burst suppression patterns, have been linked to worsened Pediatric Cerebral Performance Category scores, increased mortality in children with conditions such as electrographic status epilepticus, and unfavourable behavioral outcomes (26, 27). Understanding the baseline effects of sedatives on aEEG patterns in neurologically intact children provides an important reference point for interpreting aEEG changes in critically ill children. In conjunction with recently described normal values (28–30), it enables better differentiation between sedation-induced amplitude changes and pathological alterations. As the aEEG often serves as a bridging technology until full channel EEG becomes available or as an adjunct to continuous EEG monitoring in pediatric critical care (2, 31), this knowledge exerts direct clinical impact in daily practice.

Our study has several limitations: The exclusive use of propofol, along with the fact that the children involved were neurologically healthy and cardiorespiratory stable, limits the generalisability of our findings to paediatric intensive care settings, where patients often have multiple coexisting conditions and are subjected to various sedation regimens. Due to the risk of potentially life-threatening propofol infusion syndrome, it is not recommended for long-term sedation in children and adolescents under 16 years of age (32). However, bolus or short-term application is frequently applied for the deepening of sedation during procedures in critically ill children. Therefore, understanding of aEEG changes typically induced by propofol are valuable for paediatric critical care providers. Our study has two more limitations that are related: the device’s design for intraoperative neuromonitoring limited recordings to frontal channels only, and these frontal channels are particularly prone to artifacts.

## 5 Conclusion

In conclusion, this study enhances understanding of how propofol sedation affects aEEG in neurologically healthy children, revealing age-related and sedation depth-related changes. Our findings underscore the potential for aEEG to aid in monitoring sedation levels. However, aEEG is probably not suitable as the sole method to assess sedation depth. Anesthesia monitoring systems integrate frequency-based information and other EEG features to better detect over- or under-sedation, which aEEG alone cannot provide reliably. Nonetheless, the broad availability and bedside usability of aEEG justify its use as an adjunct, especially in resource-limited settings or when full EEG is delayed. Even though the results from this study are limited to stable children, they offer some insights for guiding sedation management in paediatric critical care. Further research is needed to explore the applicability of aEEG monitoring in more complex paediatric patients.

## Data Availability

The raw data supporting the conclusions of this article will be made available by the authors, without undue reservation.
